# Oxidative Degradation of Methylene Blue via PDS-Based Advanced Oxidation Process Using Natural Pyrite

**DOI:** 10.3390/ijerph16234773

**Published:** 2019-11-28

**Authors:** Liang Sun, Dehao Hu, Ziyu Zhang, Xiaoyan Deng

**Affiliations:** College of Environment and Safety Engineering, Qingdao University of Science and Technology, Qingdao 266042, China; hudehaoqust@126.com (D.H.); a3360909024@outlook.com (Z.Z.); dengxiaoyanqust@126.com (X.D.)

**Keywords:** advanced oxidation process, pyrite, sulfate radical, methylene blue

## Abstract

H_2_O_2_- and PDS-based reactions are two typical advanced oxidation processes (AOPs). In this paper, a comparative study of H_2_O_2_/PDS-based AOPs employing natural pyrite as a catalyst to degrade methylene blue (MB) was reported. The adaptive pH range in pyrite/PDS extended from 3 to 11, in contrast to the narrow effective pH range of 3–7 in pyrite/H_2_O_2_. As a result of the iron leaching, a synergistic effect of both homogeneous and heterogeneous catalysis was observed in pyrite/PDS, whereas heterogeneous catalytic oxidation dominated pyrite/H_2_O_2_. Furthermore, the batch results showed that the MB removal by pyrite/PDS was highly dependent on chemical conditions (e.g., pH, pyrite and PDS concentration, temperature). Powerful SO_4_^•−^ was generated by pyrite rapidly under acidic or weakly acidic conditions, while SO_4_^•−^ and PDS were assumed by OH^−^ under alkaline condition. The lower pyrite loading (from 0.1 to 0.5 g/L) was affected the removal efficiency obviously, while the scavenging of SO_4_^•−^ did not seem to be remarkable with the excessive amounts of pyrite (>0.5 g/L). Excessive amounts of PDS (>2 mmol/L) might negatively affect the pyrite/PDS system. The reaction temperature that increased from 20 to 40 °C had a positive effect on the degradation of MB. SEM and XRD showed that the passivation of catalyst did not occur due to the strong acid-production ability of pyrite/PDS, inhibiting the formation of Fe-oxide covering the pyrite surface.

## 1. Introduction

Many industries discharge organic dyes into the natural environment during synthesis and processing [1,2,3]. As one kind of dyes, methylene blue (MB) is widely used in industrial processes, such as textiles, printing, paper, pharmaceuticals, and cosmetics processing [4]. As a toxic contaminant, MB is toxic, carcinogenic, and non-biodegradable, which can cause a serious threat to environmental safety and human health [5]. Additionally, this kind of pollutant has remarkable antibiotic effects on microorganisms in traditional biological treatment processes. Therefore, it is important to explore a practical method for improving the degradation of MB from water pollution. 

Various physical and chemical treatment techniques, including photo-catalysis [6], adsorption [7], coagulation [8], chemical oxidation [9], have been investigated to effectively remove MB. Among them, advanced oxidation processes (AOPs) have shown great potential in advanced treatment due to the high removal efficiency of refractory compounds [10,11,12]. As one kind of AOP, the Fenton process has been proved to be feasible because of its high efficiency, simple operation, and low cost [13,14,15]. However, in spite of the high oxidation performance, the classic Fenton reaction (which is catalyzed by soluble Fe^2+^) has some critical limitations: the operation needs to start under low initial pH (the optimum pH usually is 3) and the stoichiometric quantity of soluble Fe^2+^ that must be added generates significant amounts of sludge [16,17]. To overcome these drawbacks mentioned above, various modified methods have been developed, including the use of chelated iron, photo-Fenton, electro-Fenton, and heterogeneous catalyst [18,19]. 

For the past years, researchers have found that sulfate radical (SO_4_^•−^)-based advanced oxidation processes are promising advanced oxidation technologies, which use SO_4_^•−^ as the reactive radical species to degrade pollutants [20,21]. Compared with HO⋅, SO_4_^•−^ radicals have higher oxidation potentials (2.5–3.1 V), longer half-life (30–40 μs) and higher selectivity to contaminants with unsaturated or aromatic rings [22]. Additionally, SO_4_^•−^ are reported to be less susceptible to common inorganic ions in aquatic environments and can maintain high-oxidation properties from acidic to alkaline conditions [23,24,25]. Na_2_S_2_O_8_ (PDS) is a typical precursor to generate SO_4_^•−^, and transition metal ions, such as Fe^2+^, Co^2+^, Cu^2+^, and Ag^+^ have been used to activate PDS [26]. Among them, iron is widely used for its cost-effective, eco-friendly, and efficient properties as Equations (1) and (2) [27]:

(1) $$Fe^{2+}+S_{2}O_{8}^{2-}\to Fe^{3+}+SO_{4}^{•-}+SO_{4}^{2-}$$

(2) $${\mathrm{Fe}(\mathrm{II})+S}_{2}O_{8}^{2-}\to Fe(III)+SO_{4}^{•-}+SO_{4}^{2-}$$

Pyrite (FeS_2_) is the most abundant metal sulfide on the surface of Earth and can be an appropriate material to act as a heterogeneous catalyst in the Fenton reaction [28]. Bae et al. [29], for instance, observed that the pyrite-catalyzed Fenton process was very effective in treating diclofenac. Che et al. [30] showed that the Fenton process using pyrite suspension effectively degraded trichloroethylene under a wide pH range. Our previous study also developed an enhanced Fenton system catalyzed by natural pyrite to degrade actual chemical wastewater [28]. 

As stated above, although pyrite has been used as the catalyst in Fenton treatment of several organic contaminants, an extensive comparative study regarding the application of PDS-based AOPs employing natural pyrite as a catalyst is not available in the literature. It is necessary to compare the removal performance between the pyrite/H_2_O_2_ and pyrite/PDS under acidic to alkaline conditions. The underlying reason causing the different applicability of these two typical AOPs remains an interesting problem to be solved. Additionally, data are needed to understand the effects of chemical conditions (e.g., pH, pyrite loading, PDS concentration, and temperature) on pyrite/PDS process efficiency so that the feasibility and application of this technology can be evaluated.

In the present study, methylene blue was selected as the target pollutant because it is one of the most widely used organic dye and has been reported as a typical contaminant with aromatic rings pollutant occurring in aquatic environments. The purpose of this current work is to: (1) determine the different pH applicability among pyrite/H_2_O_2_ or pyrite/PDS and further confirm the underlying reason; and (2) better understand the effects of certain chemical parameters such as pH, pyrite loading, PDS concentration, and temperature on MB removal by pyrite/PDS in batch systems. In addition, the possible surface characterizations of pyrite before and after oxidation are also proposed. The results contribute to a better understanding of the mechanism and reaction process of the pyrite/PDS.

## 2. Materials and Methods 

### 2.1. Materials

Chemicals used in the experiments consisted of reagent grade (AR) methylene blue (MB), Na_2_S_2_O_8_ (PDS), H_2_SO_4_ and NaOH were obtained from the Sinopharm Chemical Reagent Co. Ltd., Shanghai, China. The pyrite used in the experiments was mined from Anhui, China. The pyrite was sieved to a 300-mesh powder, washed with 1 mol HCl to remove surface oxidation layers, rinsed three times with deoxygenated deionized water and dehydrated with ethanol, and dried and stored in a closed vial under a pure nitrogen atmosphere. Elemental analyses showed that the iron content of the pyrite was 25%. The BET surface area of the pyrite was 5.960 m^2^/g.

### 2.2. Experimental Procedures

The performance of pyrite/PDS was investigated by batch experiments. In each batch experiment, 200 mL of the desired concentration of MB (100 mg/L) solution was contained with appropriate amounts of pyrite under certain concentrations of H_2_O_2_ or PDS. The pH was then adjusted by NaOH or H_2_SO_4_. The reaction began once the magnetic stirring was maintained at the same speed (150 r/min) in the experiments. At predetermined time intervals, 2 mL of the reaction sample was withdrawn and immediately mixed with 10 μL methanol to terminate the reaction. All experiments were conducted in triplicate.

### 2.3. Analytical Methods

All samples were filtered through 0.45 µm polyethersulfone filters (ANPEL Scientific Instrument Co. Ltd., Shanghai, China). The concentration of MB was examined by a PELambda 25 UV–VIS light spectrophotometer (at 664 nm) (Yuanxi, Shanghai, China). Total iron concentration was determined with the atomic absorption spectrophotometer (MSERIES ICE, Thermo Scientific, Waltham, MA, USA). Ferrous iron was measured according to the 1,10-phenanthroline method [15]. The pH was measured with a redox combination electrode (Thermo Scientific, Dual Star, Waltham, MA, USA). The pyrite samples before and after reactions were characterized by SEM (Nova NanoSEM450, 20 kV, Thermo Fisher Scientific, Shanghai, China), and XRD (Rigaku D/Max-2550, Kyoto, Japan).

## 3. Results and Discussion

### 3.1. Performance of MB Degradation in a Pyrite/H_2_O_2_ or Pyrite/PDS System

**Figure 1:**
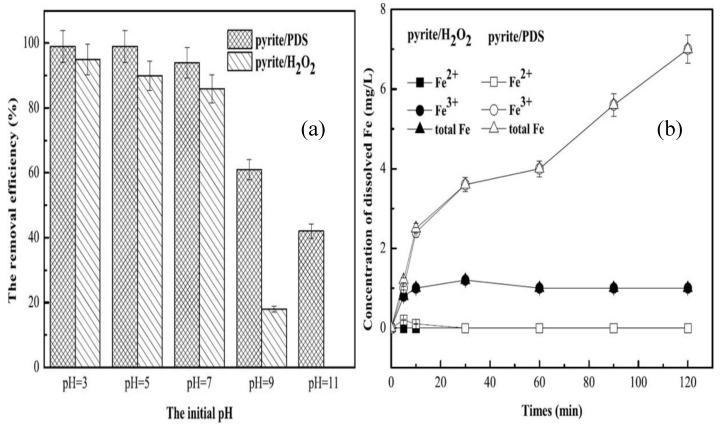
Degradation of MB in pyrite/PDS and pyrite/H_2_O_2_ system: (**a**) the removal efficiencies after 120 min; (**b**) the concentration of dissolved Fe. (Experimental conditions: [MB]_0_ = 100 mg/L, [pyrite] = 0.5 g/L, [PDS] = [H_2_O_2_] = 2 mmol/L, initial pH = 5, 25 °C).

Performance of MB degradation in pyrite/H_2_O_2_ and pyrite/PDS system were both obtained under 0.5 g/L pyrite, 2 mmol/L oxidant and different desired initial pH. We compared the removal efficiency between the pyrite/PDS and the pyrite/H_2_O_2_ (Figure 1a). The results showed that the removal efficiencies were similar whether an acidic condition or neutral condition (more than 90%). When the initial pH was 9, the removal efficiency of pyrite/PDS was 61%, whereas that of pyrite/H_2_O_2_ was decreased sharply to 18%, additionally, when the initial pH was 11, there was no MB removal in pyrite/H_2_O_2_ process, while the removal efficiency of pyrite/PDS still reached 42%.

The degradation kinetics of MB in pyrite/H_2_O_2_ or pyrite/PDS was properly fitted by a pseudo-first-order rate law. The kinetic constant of the reaction was summarized in Table 1. When the initial pH was acidic or neutral, the k*_obs_* of pyrite/PDS were more than two times those with pyrite/H_2_O_2_, respectively. However, when the initial pH increased to 11, the k*_obs_* of pyrite/PDS decreased sharply to 0.0037 min^−1^, in contrast, the k*_obs_* of pyrite/H_2_O_2_ was not calculated because of the negative removal efficiency.

It is clear that pyrite/PDS was applied to a wider pH range than pyrite/H_2_O_2_. The phenomenon was assumed to the following reasons: previous studies have illustrated that HO⋅ was generated by the activation of H_2_O_2_ via pyrite (Equations (3) and (4), while SO4^•−^/HO⋅are the products after PDS activation (Equations (5)–(9) [30,31,32,33,34]. As a heterogeneous material, pyrite can expand the effective pH range of the Fenton process successfully. When the initial pH was at neutrality, the pH first decreased drastically from 7 to 5.5, and then gradually decreased to pH 4.8 in our experiments. Importantly, the decreased pH helped sustain the HO⋅ generation. However, when the initial pH was alkaline, the mass of OH^−^ inhibited the generation of HO⋅ according to Equations (3) and (4), and the pH was still alkaline during the whole process. For the pyrite/PDS, pyrite can easily activate PDS to generate SO_4_^•−^ due to Equations (5) and (6) under acidic or neutral conditions. When the initial pH was alkaline, Furman et al. pointed out that PDS rapidly decomposed to hydroperoxide and sulfate by OH^−^. Then, the hydroperoxide formed from the hydrolysis of one persulfate molecule reduces another persulfate molecule, generating SO4^•−^ (Equations (7) and (8)). Furthermore, SO_4_^•−^ can react with OH^−^ to form HO⋅ (Equation (9)) [35]. In a word, the cause of the less efficient oxidation of pyrite/H_2_O_2_ under alkaline conditions is entangled in the lack of generated HO⋅, while different mechanisms of SO4^•−^ generation account for the efficient removal of pyrite/PDS from acidic to alkaline conditions.

To explain the difference of the MB removal for pyrite/H_2_O_2_ (0.00157 min^–1^) and pyrite/PDS (0.00332 min^–1^) under the same initial pH (the initial pH = 5), we monitored the variation of iron during the process of pyrite/H_2_O_2_ and pyrite/PDS. As shown in Figure 1b, the total iron concentration produced in pyrite/PDS (7 mg/L) was much higher than that in pyrite/H_2_O_2_ (1 mg/L), indicating that PDS more easily reacted with pyrite. In addition, Fe^3+^ was the dominant iron ion in both pyrite/PDS and pyrite/H_2_O_2_. The results showed that Fe^2+^ dissolved from the pyrite surface was quickly oxidized to Fe^3+^ and used for the formation of HO⋅ or SO4^•−^ in the pyrite AOPs system. Chou and Bae et al. showed that a significant homogeneous promotion effect occurred only when the concentration of dissolved iron species was above 3.92 mg/L [29,36]. For pyrite/H_2_O_2_, the lack of dissolved iron only weakly promoted the catalytic oxidation of MB, while the homogeneous promotion effect was not ignored in pyrite/PDS because of the high iron leaching. To verify this conclusion, the removal efficiencies of MB by Fe^2+^ (7 mg/L)-activated PDS were determined. The removal efficiency reached to 32.6% after 120 min, suggesting that a synergistic effect of both homogeneous and heterogeneous catalysis occurred and Both Fe^2+^ (Equation (6)) and SO_4_^•−^ (Equation (5)) generating reactions are major rate-limiting steps.

In fact, although the adaptive pH range of pyrite/PDS is wider than that of pyrite/H_2_O_2_, it is difficult to draw a definite conclusion that which AOPs is better because it needs an assessment system, which involved the best working pH, oxidation efficiency, increment of sulfate ions, and the toxicity after oxidation.

(3) $${\mathrm{Fe}}^{\mathrm{ΙΙ}}S_{2}{+H}_{2}O_{2}\to Fe^{\mathrm{ΙΙΙ}}S_{2}+OH^{-}+HO\cdot$$

(4) $${\mathrm{Fe}}^{2+}{+H}_{2}O_{2}\to Fe^{3+}+OH^{-}+HO\cdot$$

(5) $$Fe^{\mathrm{ΙΙ}}S_{2}+S_{2}O_{8}^{2-}\to Fe^{\mathrm{ΙΙΙ}}S_{2}+SO_{4}^{•-}+SO_{4}^{2-}$$

(6) $${\mathrm{Fe}}^{2+}+S_{2}O_{8}^{2-}\to Fe^{3+}+SO_{4}^{•-}+SO_{4}^{2-}$$

(7) $$S_{2}O_{8}^{2-}+2H_{2}O\overset{OH^{-}}{\to }HO_{2}^{-}+2SO_{4}^{2-}+3H^{+}$$

(8) $$S_{2}O_{8}^{2-}+HO_{2}^{-}\overset{OH^{-}}{\to }SO_{4}^{•-}+SO_{4}^{2-}+H^{+}+O_{2}^{•-}$$

(9) $$SO_{4}^{•-}+OH^{-}\to HO\cdot +SO_{4}^{2-}$$

where Fe^2+^ and Fe^3+^ are the dissolved iron, Fe^II^ and Fe^III^ represent the surface Fe(II) or Fe(III) of pyrite.

### 3.2. Effect of Initial pH on MB Removal by Pyrite/PDS

**Figure 2:**
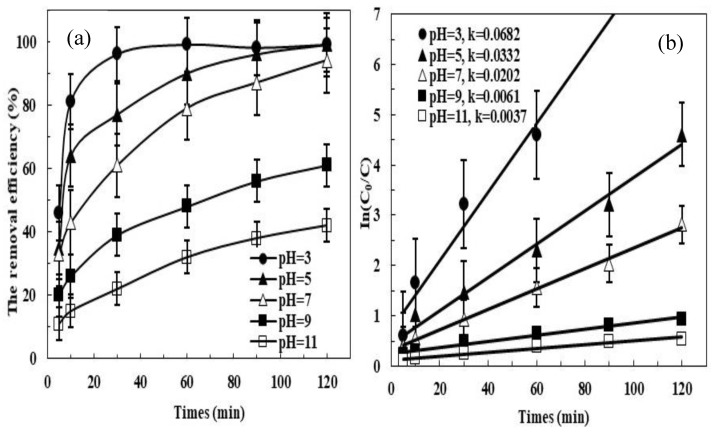
Effect of initial pH on the MB degradation by pyrite/PDS process: (**a**) the removal efficiency; (**b**) In(C_0_/C). Experimental conditions: [MB]_0_ = 100 mg/L, [pyrite] = 0.5 g/L, [PDS] = 2 mmol/L, 25 °C.

The pH is the main factor significantly influencing the degradation performance. To evaluate thoroughly the effect of the initial pH, batch experiments with different initial pH (3, 5, 7, 9, and 11) were set up by adding suitable materials. As shown in Figure 2a, the removal efficiency was 99% after 60 min when the initial pH was 3 whereas it reached more than 90% after 120 minutes under neutral condition. Meanwhile, the removal efficiency was decreased with the increased initial pH under alkaline condition. It was 61% when the initial pH was 9 and it was 42% when the initial pH was 11. Figure 2b shows that the k*_obs_* decreased from 0.0682 min^−1^ to 0.0332 min^−1^ with an increase initial pH from 3 to 5, then the values began to decrease sharply with the continuous increase initial pH (from 0.0332 min^−1^ to 0.0037 min^−1^).

Kantar et al. found that the decrease of removal efficiency with increasing solution pH may be attributed to the decrease in iron (III) solubility. For the pyrite/H_2_O_2_, solid-phase iron oxide or iron hydroxide can accumulate on pyrite surface due to the high pH, inhibiting the generation of HO⋅ [37]. For the pyrite/PDS system, the reaction between PDS and pyrite was an acid-production process (according to Equation (10)), which was beneficial for the oxidation reaction. We observed that initial pHs (5, 7, and 9) rapidly decreased to 3.7, 4.1, and 5.8 immediately after the addition of PDS, respectively, and reached below 3.5 in 40 min. When the initial pH was 11, the pH first decreased drastically to 8.73, and then gradually decreased to pH 4.8. Low pH conditions inhibit the precipitation of Fe^2+^ and Fe^3+^ hydroxide complexes such as Fe(OH)_2_, Fe_2_(OH)_2_^4+^, and Fe(OH)_3_, whose catalytic activities for SO_4_^•−^ generation are much lower than that of pyrite [38]:

(10) $$2\mathrm{Fe}S_{2}+15S_{2}O_{8}^{2-}+16H_{2}O\to 2Fe^{3+}+34SO_{4}^{2-}+32H^{+}$$

The k*_obs_* at initial pH 3 (0.0682 min^−1^) and 5 (0.0332 min^–1^) was more than 10 times higher than that at pH 11 (0.0037 min^−1^). The results showed that PDS can be activated to generate powerful SO_4_^•−^ rapidly under acidic or weak acidic condition, whereas under alkaline conditions the SO_4_^•−^ and PDS were eliminated according to Equations (11) and (12), which resulted in the decline of the removal efficiency and apparent rate constants of reactions:

(11) $$SO_{4}^{•-}+{\mathrm{OH}}^{-}\to HSO_{4}^{-}+1/2O_{2}$$

(12) $$S_{2}O_{8}^{2-}+H_{2}O\to 2HSO_{4}^{-}+1/2O_{2}$$

### 3.3. Effect of Pyrite Loading and PDS Dosage on MB Removal by Pyrite/PDS 

**Figure 3:**
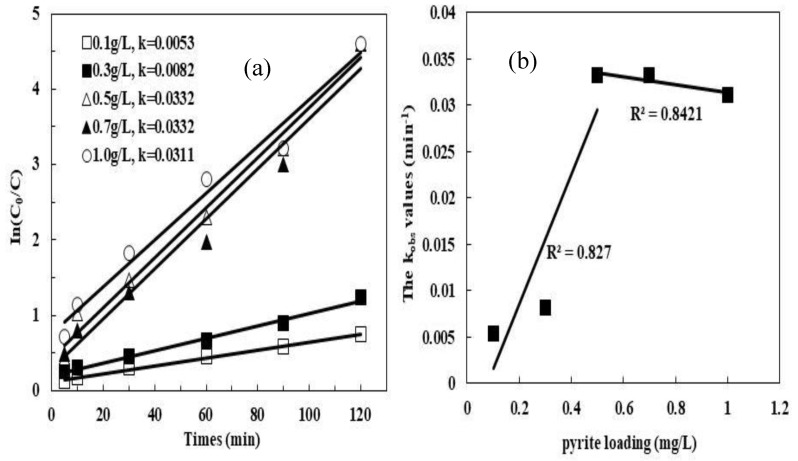
Effect of the loading of catalysts on the MB degradation by pyrite/PDS process: (**a**) In(C_0_/C); (**b**) change of kinetic rate constants with respect to pyrite loading. Experimental conditions: [MB]_0_ = 100 mg/L, [PDS] = 2 mmol/L, initial pH = 5, 25 °C.

The effect of natural pyrite dosage on the removal of MB was examined in batch experiments using different initial dosages (0.1–1 g/L). Figure 3 shows the effect of pyrite loading. Within 120 min, the k*_obs_* was 0.0053, 0.0082, 0.0332, 0.0332, and 0.0311 min^–1^, which correspond to the pyrite dosage of 0.1, 0.3, 0.5, 0.7, and 1.0 g/L, respectively. The increase of kinetic rate constants with pyrite loading can be explained by a linear regression fit (Figure 3b) of which slope under the pyrite loading range is from 0.1 to 0.5 g/L, then the change of k*_obs_* was not obvious with the continuous increase pyrite loading (>0.5 g/L).

Pyrite surface site concentration (SOH) was estimated to be 23.03 μmol/g based on pyrite surface area of 5.960 m^2^/g as follows [39]:

(13) $$SOH_{T}=\frac{S_{A}\eta_{s}10^{18}}{N_{A}}$$

where S_A_ is the surface area (m^2^/g), N_A_ represents Avogadro’s number and n_s_ is the average site density (2.33 sites/nm^2^). The surface site concentration for 0.5 g/L pyrite is estimated to be 11.515 μmmol. Note that the surface site concentrations are much less than the MB concentrations (100 mg/L evaluated as 267.45 μmol/L) used in the experiments, implying that processes involving both solution and surface phase reactions occurred very fast during pyrite/PDS. Previous authors reported that two types of oxidation occur during the pyrite/PDS process: (1) pyrite oxidation and SO_4_^•−^ oxidation [21,22]. Increasing the loading of pyrite proportionally increased the aqueous Fe^2+^ concentration, which improved SO_4_^•−^ formation and enhanced the degradation of MB. Furthermore, Fe^2+^ generating reaction is one of the major rate-limiting steps. As the pyrite loading increased, the generation of Fe^2+^ can be enhanced due to the reduction of Fe^3+^ on the increased pyrite surface (Equation (14)) [28]:

(14) $$FeS_{2}+14Fe^{3+}+8H_{2}O\to 15Fe^{2+}+2SO_{4}^{2-}+16H^{+}$$

Previous authors pointed out that an excessive amount of aqueous Fe^2+^ may promote the unwanted consumption of SO_4_^•−^ (Equation (14) [28,31,32], which can negatively affect the oxidative degradation. Interestingly, the scavenging of SO_4_^•−^ in pyrite/PDS did not seem to be remarkable with the increase of pyrite loading. This may be due to the low aqueous Fe^2+^ generation:

(15) $$Fe^{2+}+SO_{4}^{•-}\to Fe^{3+}+SO_{4}^{2-}$$

**Figure 4:**
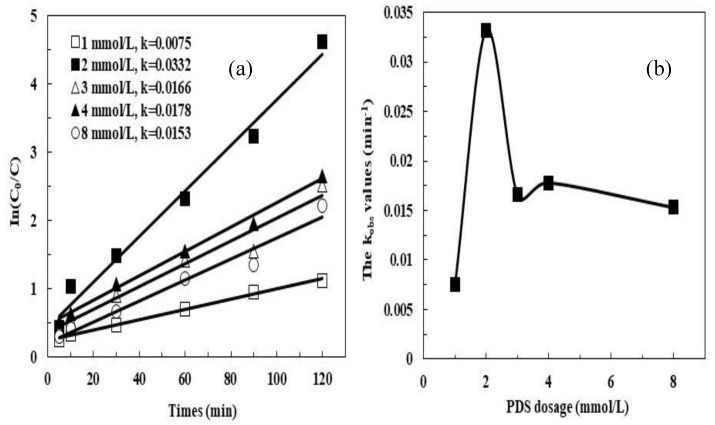
Effect of PDS dosage on the MB degradation by the pyrite/PDS process: (**a**) In(C_0_/C); (**b**) change of kinetic rate constants with respect to PDS dosage. Experimental conditions: [MB]_0_ = 100 mg/L, [pyrite] = 0.5 g/L, initial pH = 5, 25 °C.

Figure 4 shows the effect of the initial PDS concentration on the removal of MB. When the initial PDS was 1 mmol/L, the k*_obs_* was 0.0075 min^–1^ after 120 min, but at an initial PDS concentration of 2 mmol/L, the k*_obs_* increased to 0.0332 min^–1^. However, when the initial PDS concentration increased to 8 mmol/L, the k*_obs_* value decreased to 0.0153 min^–1^. These variations were consistent with those observed in previous studies, which proposed that when an appropriate concentration of Fe^2+^ was provided in the reaction system, an increase of the PDS concentration can enhance the oxidative degradation of organics due to the improvement in SO_4_^•−^ formation. In contrast, an excessive amount of PDS readily reacts with generated SO_4_^•−^ (Equation (16)) [32]:

(16) $$SO_{4}^{•-}+S_{2}O_{8}^{2-}\to S_{2}O_{8}^{•-}+SO_{4}^{2-}$$

### 3.4. Effect of the Reaction Temperature on the Catalytic Performance

**Figure 5:**
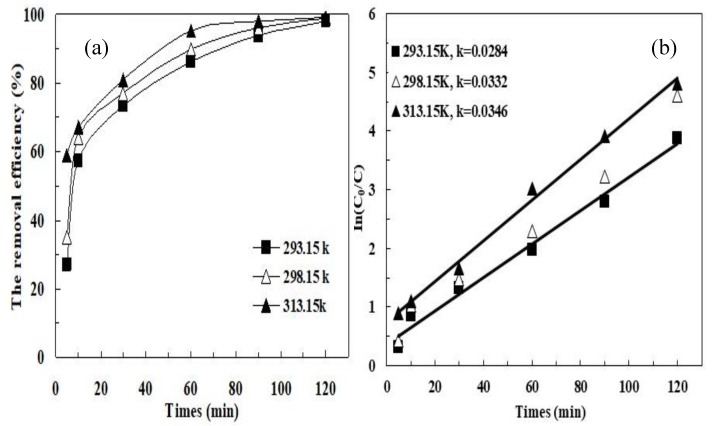
Effect of reaction temperature on the MB removal by the pyrite/PDS process:(**a**) the removal efficiency; (**b**) In(C_0_/C). Experimental conditions: [MB]_0_ = 100 mg/L, [pyrite] = 0.5 g/L, [PDS] = 2 mmol/L, initial pH = 5.

The effect of temperature on MB degradation was investigated at 20 °C, 25 °C, and 40 °C under optimal conditions. The results showed that increasing temperature had a positive effect on the degradation of MB. As shown in Figure 5a, the degradation of MB after 90 min was 93.9% at 20 °C, while 90% and 95.1% degradation was achieved at 25 °C and 40 °C after 60 min, respectively. Figure 5b shows the MB removal by pyrite/PDS with different reaction temperatures all followed the pseudo-first-order kinetics. The k*_obs_* increased from 0.0284 min^−1^ to 0.0346 min^−1^ when the reaction temperature increased from 20 °C to 40 °C. Interestingly, previous work showed that PDS can be activated by heat [40]. A set of control experiments were also conducted to investigate the degradation of MB by PDS without pyrite at the same temperatures. After 120 min, the removal efficiencies increased from 15.9% to 42.2% when the reaction temperature increased from 20 °C to 40 °C in a PDS-only system. Based on the MB degradation in these two systems, reaction temperature significantly promoted SO_4_^•−^ generation catalyzed by pyrite. Activating persulfate by increasing temperature might not be a promising way because the removal performance was not enhanced notably until under high reaction temperature such as 80 °C [40]. It is difficult to heat the wastewater to high temperature and too much energy should be consumed. Therefore, the application of pyrite is a better way to activate persulfate because the pyrite/PDS is effectively controlled by the reaction temperature. 

### 3.5. Surface Characterization of Pyrite Using in Pyrite/PDS Process

**Figure 6:**
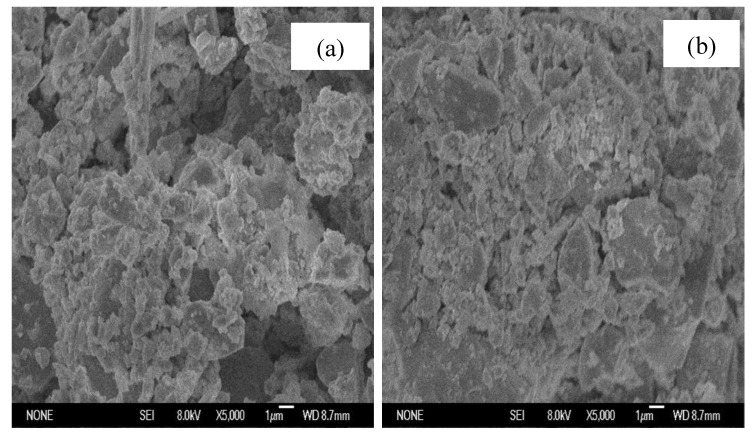
The SEM of the pyrite: (**a**) the natural pyrite; (**b**) the pyrite after reaction. Experimental conditions: [MB]_0_ = 100 mg/L, [pyrite] = 0.5 g/L, [PDS] = 2 mmol/L, initial pH = 5, 25 °C.

**Figure 7:**
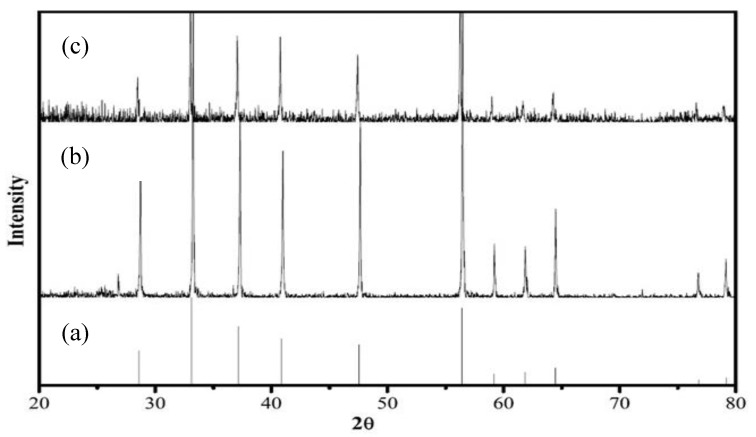
Comparison of XRD of pyrite in different periods: (**a**) pyrite standard card (FeS_2_-PDF#89-3057); (**b**) the initial pyrite; (**c**) the pyrite after reaction. (Experimental conditions: [MB]_0_ = 100 mg/L, [pyrite] = 0.5 g/L, [PDS] = 2 mmol/L, initial pH = 5, 25 °C).

The surface morphology and crystal structure of the prepared natural pyrite before and after the PDS oxidation process were observed by SEM and XRD, respectively. Figure 6a shows the SEM image of raw pyrite, whereas Figure 6b show the SEM image of the used pyrite. The comparison of XRD of pyrite in different periods is shown in Figure 7. 

The SEM image of natural pyrite had a messy floc structure (Figure 6a). Figure 6b shows the SEM images of the pyrite after the reactions which acted as a dense and cluster substance. According to the pyrite standard card (Figure 7a), it was clear that only the phase of pyrite was detected and there was no other phase such as Fe_2_O_3_ or Fe_3_O_4_ (Figure 7b). In addition, crystalline phases of catalyst did not change significantly after the reactions (Figure 7c). 

The Eh-pH simulations showed that the possible oxidized pyrite surface species include hematite (Fe_2_O_3_), goethite (FeOOH) and ferrihydrite (Fe(OH)_3_) under slightly acidic to alkaline pH conditions in pyrite/H_2_O_2_ [41] and Oral et al. confirmed the conclude and illustrated that the accumulation of solid-phase iron oxide or iron hydroxide inhibited the generation of HO⋅ [31]. However, we did not find a similar phenomenon in pyrite/PDS (Figure 7). Passivation did not occur in pyrite/PDS because of the following reasons: (1) the dissolved SO_4_^2–^, which was the main product during the reactions between PDS and pyrite, was unable to attach to the surface of pyrite; and (2) acidity was produced to inhibit the formation of Fe-oxide covering the pyrite surface. 

Additionally, regeneration of Fe(II) by S_2_^2–^ on the pyrite surface in pyrite/PDS has not been explored in this paper. Zhou et al. showed that S_2_^2–^ was the dominant electron donor, while Fe(II) regeneration via the reduction of Fe(III) by S_2_^2–^ on the pyrite surface was the key step controlling radical production and pollutant degradation during PMS activation by pyrite [42]. Peng et al. also found that S_2_^2–^ was beneficial to the catalytic performance of pyrite [21]. Hence, the difference in the relative content of sulfur species and sulfur conversion on the surface of pyrite during the whole process is very interesting, and further investigation is ongoing.

## 4. Conclusions

This study suggests that the pyrite/PDS system is an effective oxidant process for MB degradation. Compared with pyrite/H_2_O_2_, pyrite/PDS can be applied to a wider pH range. Furthermore, experimental parameters affected the reactivity of pyrite/PDS, including the initial pH, pyrite and PDS loading, and the reaction temperature. Pyrite/PDS exhibited degradation activity within a pH range of 3–11. No scavenging effect was observed when using pyrite as catalyst. Temperature significantly promoted MB degradation. Additionally, the passivation of catalysts did not occur in the pyrite/PDS process. In brief, the main advantages of pyrite/PDS, therefore, summarize as: (1) multiple pathways for SO4^•^^−^ generation under different pH; and (2) stronger acid-producing ability which prevented the passivation of catalyst.

## Figures and Tables

**Table 1:** Summary of pseudo-first-order rate constants for MB degradation.

The Initial pH	Oxidant (mmol/L)	Pyrite (g/L)	Pyrite/PDS		Pyrite/H_2_O_2_	
			$k_{obs}\times 10^{2}\min^{-1}$	R^2^	$k_{obs}\times 10^{2}\min^{-1}$	R^2^
pH = 3	2	0.5	6.82	0.9444	3.21	0.9765
pH = 5	2	0.5	3.32	0.9843	1.57	0.9132
pH = 7	2	0.5	2.02	0.996	1.12	0.8358
pH = 9	2	0.5	0.61	0.9738	-	-
pH = 11	2	0.5	0.37	0.9783	-	-
